# Survival of Children Living With HIV on Art in Zambia: A 13-Years Retrospective Cohort Analysis

**DOI:** 10.3389/fpubh.2020.00096

**Published:** 2020-03-31

**Authors:** Tendai Munthali, Charles Michelo, Paul Mee, Jim Todd

**Affiliations:** ^1^School of Public Health, University of Zambia, Lusaka, Zambia; ^2^Department of Public Health, Ministry of Health, Lusaka, Zambia; ^3^MeSH Consortium, Department of Public Health Environments and Society, Faculty of Public Health and Policy, London School of Hygiene and Tropical Medicine, London, United Kingdom; ^4^Department of Population Health, London School of Hygiene and Tropical Medicine, London, United Kingdom

**Keywords:** survival, HIV- infected, children, Zambia, anti-retroviral treatment (ART), initiation

## Abstract

**Background:** Research conducted before the introduction of anti-retroviral therapy (ART), showed that the majority of children living with HIV (CLHIV) would die before their second birthday. In Zambia, ART was rolled out to the public health system in 2004 with subsequent improved survival in CLHIV. However, the survival rates of CLHIV on ART in Zambia since 2004 have not been extensively documented. We assessed survival experiences and the factors associated with survival in CLHIV on ART in Zambia.

**Methods:** We conducted a retrospective cohort analysis of CLHIV (aged up to 15 years) using routinely collected data from health facilities across Zambia, over 13 years to ascertain mortality rates. We explored survival factors using Cox regression giving adjusted hazard ratios (AHR) and 95% confidence intervals (95% CI). Nelson Aalen estimates were used to show the cumulative hazards of mortality for different levels of explanatory factors.

**Results:** A total of 65,448 eligible children, were initiated on ART between 2005 and 2018, of which 33,483 (51%) where female. They contributed a total survival time of 275,715-person years at risk during which 3,265 children died which translated into an incidence rate of 1.1 deaths per 100 person-years during the review period. Mortality rates were highest in children in the first year of life (Mortality rate 2.24; 95% CI = 2.08–2.42) and during the first year on ART (Mortality rate 3.82 95% CI = 3.67–3.98). Over 50% of the children had been on ART for 5–10 years by 2018, and they had the lowest risk of mortality compared to children who had been on ART for <5 years.

**Conclusions:** Children with HIV in Zambia are surviving much longer than was predicted before ART was introduced 14 years ago. This key finding adds to the literature on analysis of survival in CLHIV in low income settings like Zambia. However, this survival is dependent on the age at which ART is initiated and the time on ART highlighting the need to increase investments in early infant diagnosis (EID) to ensure timely HIV testing and ART initiation for CLHIV.

## Introduction

UNAIDS has a target to end all new HIV infections by 2030, but in 2018 there were still 1.7 million children living with HIV (CLHIV) (1). Worldwide, every year there are 110,000 deaths in CLHIV under the age of 15 years, and 180,000 new HIV infections from mother to child transmission (1, 2). In Zambia, in 2018 there were ~62,000 children living with HIV, 79% of them were on ART and there were 3,000 AIDS related annual deaths (3, 4). Without ART, more than 50% of children infected with HIV will die before their second birthday (5, 6). From its inception ART has been shown to reduce mortality among adults and children living with HIV more than 50% since the year 2000 (7–9). With the roll out of HIV treatment services, the initiation of CLHIV on ART in Zambia increased steadily from 24,000 in 2010 to 49,116 in 2018 (2, 3). This translated into a reduction in HIV-related mortality from 3,600 in 2016 to 3,000 in 2018 (3, 4).

However, studies show that mortality among CLHIV on ART continues to be high, especially among infants with advanced disease, and in the first 12 months following ART initiation despite the use of prevention of mother-to-child transmission of HIV (PMTCT) drugs (10, 11). Studies have shown that initiating children on ART early (birth-7 weeks) reduced mortality by 76% compared to postponing ART until progression to advanced HIV disease or CD4 count thresholds are reached (11, 12). However, other studies have shown that WHO clinical stage 3 or 4 and young age at the time of ART initiation were associated with a 5-fold increased risk of death, while absolute CD4 counts of <350 cells/mm^3^ at ART initiation and underweight (weight-for-age *Z*-score < -2) were associated with 3.6 and 3,5 times increase in mortality among CLHIV (5, 13–15). In addition, overall mortality rates among CLHIV on ART vary from country to country depending on several factors. In a 7-year study in South Africa, a mortality rate of 4.7 per 100 person-years was reported (16), whereas in Ethiopia a mortality rate of 1.2 per 100 person-years was reported in Addis Ababa and 2.1 per 100 person-years in Eastern Ethiopia after a 2 year follow up (14). Furthermore, a mortality rate of 3.0 per 100 person-years was reported in a 1 year follow up study in Nigeria (17).

Despite the varied mortality rates, CLHIV are surviving longer on ART. Studies in Asia and Africa have shown survival rates that ranged from 84 to 97% after 12 months of ART for children initiated on ART at a median age of 5–7 years. Another multi country study in Africa also reported above 90% (93%) survival rates after 2 years of follow up on ART (18). There is limited global data on long term survival of children on ART. In Zambia the long-term survival of CLHIV and clinical factors that are associated with patterns of survival have not been extensively documented. We therefore assessed the probability of survival and factors associated with mortality among CLHIV in Zambia.

## Materials and Methods

### Study Population and Sample

This study was a quantitative retrospective cohort study of CLHIV with records in the Zambian SmartCare data. SmartCare is an electronic patient monitoring system which is used in about 600 government health facilities in all districts in Zambia (19). The system records patient characteristics with a unique identification number, at the first contact with the clinic, and then records clinical information at each subsequent health facility visit. A total sampling from the SmartCare records of all children with a positive HIV result, aged 15 years and younger at the time of ART initiation, regardless of missing data during the period under review, was conducted for this analysis.

### Clinical Procedures

In the Zambian health care system, all children with a positive HIV test result and diagnosis are eligible to receive HIV care according to national ART prevention and treatment guidelines. This includes documenting medical history, physical examination, anthropometric measurements, socio demographic information, and WHO clinical stage. In addition, measurement of CD4+ T-cell counts or percentages, hemoglobin levels (HB), renal, liver function tests, and HIV viral loads are taken. Eligible children are then treated with a first-line regimen, either Nevirapine based (NVP), Efavirenz based (EFV), or Nucleoside reverse transcriptase inhibitors (NRTI). Children are then asked to return for clinical evaluation every 3 months (20, 21).

### Data Definitions and Statistical Methods

Follow up of children started at the date they initiated ART, and continued until death, or the date of censoring. The event of interest, death was coded as 1 for those who died, and 0 for those who were censored. The date of censoring (end date) contained the date of death for those that died, and the last clinic date for those who were lost to follow up, with all other children censored on 1st January 2018, or the date of their 15th birthday, whichever came first. The variable age at ART initiation was obtained by subtracting the child's date of birth from the date of ART initiation and was categorized into 5 age bands (birth to <1 year, 1–2, 3–5, 6–9, and 10–15 years). Time on ART was divided into 4 points that is at 1, at 2, at 5, and at 10 years on ART. Children's weight was used to calculate the weight for age Z-scores (WAZ) for children in the dataset using a WHO standard (22).

Categorical variables were summarized across all children as frequencies and percentages. Mortality rates were defined as the number of deaths divided by the person-years of follow-up with 95% confidence intervals (95% CI) for mortality rates defined for each subgroup of children. Nelson Aalen cumulative Hazards were used to graphically show the differences in mortality by subgroups of children. A modified Cox regression model, adjusting for possible heterogeneity at the facility level, was used to obtain hazard ratios (HR) and 95% confidence intervals (95% CI) for factors associated with mortality, with subsequent adjustment for sex, age at ART initiation, and province to obtain adjusted HR (aHR) and 95% CI. In order to model the unobserved covariates at health facility level, an additional parameter, theta, drawn from a Gamma distribution, was estimated in the modified Cox regression model, representing the frailty or variation in the log of the survival function across health facilities.

For socio-demographic variables, such as age, sex, year, health facility and province, a complete case analysis was conducted, using all the children, assuming the small amount of missing data were missing at random. The results present the crude analysis, the age-adjusted analysis and the frailty model that adjusts for the effect of clustering. For variables where a large number of children had missing data, a subset analysis on children with valid data was performed to assess the mortality risks for those variables.

## Results

A total of 70,718 children were recorded as having been initiated on ART from 496 health facilities and 71 districts across Zambia during the period under review. Of these only 65,448 were analyzed in survival analysis translating to 33,483 (51%) female, and 31,965 (49%) male.

The largest proportion of children analyzed were between 10 and 15 years and these accounted for about 25% of the sample while the smallest proportion was accounted for by children under 1 year of age at 10%. Mortality was also highest among children that initiated ART under 2 years, and lowest among children that initiated ART at older ages (10–15 years). Children that initiated ART during the period under review, were largely initiated on NRTIs (47%) and NVP based regimen (29%) (Table 1).

**Table 1:** Background characteristics of CLHIV on ART in Zambia from 2004 to 2018.

**Characteristic**	**Child status**		**Person years of follow up**	**Mortality rates (95% CI)**
	**Number of children that died**	**Total number of children analyzed**		
Overall	3,265	65,448 (100)	275,715	0.011 (0.011–0.012)
**Sex**				
Female	1,520	33, 483 (51)	138,737	0.80 (0.65–0.99)
Male	1,745	31,965 (49)	137,045	1.08 (0.90–1.30)
**Age when starting ART**				
Birth – <1 year	675	6,311 (10)	30,000	2.24 (2.08–2.42)
1–2 years	1,211	14,925 (23)	73,864	1.63 (1.54–1.73)
3–5 years	432	12,073 (18)	65,278	0.66 (0.60–0.791)
6–9 years	513	15,479 (23)	70,671	0.72 (0.66–0.79)
10–15 years	431	16,496 (25)	35,687	1.20 (1.09–1.32)
**WHO clinical staging**				
Stage 1	920	39,886 (71)	186,156	0.49 (0.46–0.52)
Stage 2	334	5,499 (10)	22,216	1.50 (1.35–1.67)
Stage 3	1,150	8,406 (15)	32,032	3.38 (3.38–3.80)
Stage 4	425	1,961 (4)	7,267	5.84 (5.31–6.43)
**CD4 count**				
Normal	49	1,362 (30)	7,779	0.62 (0.47–0.83)
Low	53	3,246 (71)	13,612	1.12 (0.95–1.31)
**Weight for age Z-score**				
Normal	130	5,261 (93)	37,455	0.341 (0.29–0.41)
Moderate	29	195 (3)	1,101	2.63 (1.82–3.78)
Severe	18	145 (3)	760	2.36 (1.49–3.75)
**ART regimen**				
NRTI	601	7,314 (47)	23,700	1.38 (0.78–2.44)
EFV Based	137	3,580 (23)	15,471	1.19 (0.66–2.15)
NVP based	418	4,393 (29)	8,683	4.11 (2.68–6.30)
**Year of ART initiation**				
2004/2006	473	6,035 (9)	35,904	1.31 (1.20–1.44)
2007/2009	1,243	16,445 (25)	93,977	1.32 (1.25–1.39)
2010/2012	1,003	19,026 (29)	87,213	1.15 (1.08–1.22)
2013/2015	470	17,549 (26)	51,629	0.91 (0.83–0.99)
2016/2017	76	6,393 (10)	6,991	1.08 (0.86–1.36)
**Time on ART**				
Under 1 year	2,265	11,276 (17)	59,206	3.82 (3.67–3.98)
At 1 year	430	7,856 (12)	50,211	0.85 (0.77–0.94)
At 2 years	445	22,132 (33)	104,156	0.42 (0.38–0.46)
At 5 years	123	21,614 (33)	59,543	0.20 (0.17–0.24)
At 10 years	2	2,570 (4)	2,598	0.07 (0.01–0.30)
**Time from testing to ART initiation**				
Same day	1,075	21,307 (33)	86,961	1.23 (1.16–1.31)
1 week	240	3,712 (6)	15,426	1.55 (1.37–1.76)
Up to 1 month	849	13,471 (21)	56,774	1.49 (1.39–1.59)
More than a month	1,101	26,958 (41)	116,553	0.94 (0.89–1.00)

### Health Facility and Provincial Characteristics of CLHIV on ART

Out of the three levels of health facilities that were analyzed, hospitals and health centers accounted for the highest number of person years of follow-up (140,270 and 133,904, respectively) with health posts having the lowest person years of follow up (402). Out of the 3,265 children that died, hospitals in the country accounted for about 1,542 deaths translating to about 47% of deaths recorded during the reporting period. On the other hand, facilities in Lusaka (812, 24%), Copperbelt (583, 18%) and Southern (467, 14%) provinces had the highest number of deaths amongst all the ten provinces while facilities in Muchinga province had the lowest number of deaths with 81 deaths (2.5%). Children in Muchinga, Luapula and North western provinces also had the lowest chances of survival compared to children in Central province (North western, aHR 2.05 95% CI = 1.69–2.4; Muchinga, aHR1.95 95% CI = 1.50–2.50; Luapula, aHR 1.60 95% CI = 1.30–1.95) (Table 2).

**Table 2:** Hazard ratios for CLHIV on ART by province and facility types in Zambia.

**Characteristic**	**Number of Health facilities**	**Person years of follow up**	**Number of deaths**	**Mortality rates per 100** **(95% CI)**	**Unadjusted Hazard ratios** (CI)^*^	**Adjusted Hazard Ratios** (CI)^**^
Total	404	275,715	3,265	0.011 (0.011–0.12)	–	–
**Province**						
Central	41	24,921	207	0.83 (0.72–0.95)	1	1
Luapula	26	10,753	174	1.61 (1.39–1.87)	1.78 (1.45–2.17)	1.60 (1.30–.95)
Copperbelt	98	63,448	603	0.95 (0.87–1.02)	1.05 (0.92–1.23)	1.11 (0.95–1.31)
Eastern	41	25,186	376	1.49 (1.34–1.65)	1.51 (1.27–1.79)	1.58 (1.33–1.88)
Lusaka	68	64,830	797	1.22 (1.14–1.31)	1.25 (1.07–1.46)	1.36 (1.17–1.59)
Muchinga	14	3,814	81	2.12 (1.70–2.64)	1.84 (1.42–2.38)	1.95 (1.50–2.50)
North western	22	10,938	205	1.87 (1.63–2.14)	2.06 (1.70–2.50)	2.05 (1.69–2.49)
Northern	28	8,963	145	1.61 (1.37–1.90)	1.55 (1.25–1.91)	1.56 (1.26–1.93)
Southern	101	41,190	462	1.12 (1.02–1.22)	1.26 (1.07–1.48)	1.28 (1.10–1.51)
Western	60	21,667	215	0.99 (0.86–1.13)	1.16 (0.96–1.40)	1.18 (0.97–1.42)
**Facility type**						
Hospital	103	140,270	1,542	1.09 (1.04–1.15)	1	1
Health center	292	133,904	1,709	1.27 (1.21–1.33)	1.01 (0.95–1.09)	1.07 (0.98–1.15)
Health post	6	402	2	0.49 (0.12–1.98)	0.25 (0.06–1.01)	0.27 (0.06–1.11)
**District type**						
Rural	522	237,321	2,770	1.16 (1.12–1.21)	1	1
Urban	239	38,394	495	1.28 (1.18–1.40)	1.14 (1.03–1.25)	1.08 (0.97–1.20)

* Tested using Cox proportional Hazards ratio.

** *Adjusted for adjusted for age at ART start, sex and province*.

### Survival of CLHIV on ART and Associated Factors

A total of 65,448 children were analyzed using proportional hazards survival analysis, with a total of 275,715 person-years at risk. The individual children had follow-up times that ranged from 1 month to 13.5 years. Survival of children on ART was highest among children who initiated ART between 10 and 15 years, with these children having 72% lower hazard ratio compared to children initiating ART between birth and 1 year (aHR 0.28,95% CI = 0.25–0.32). There was considerable variability in survival amongst the children initiating ART from the different provinces (Table 2). Factors associated with mortality included age when initiating ART and time on ART although these two variables were correlated as only children who initiated ART at younger ages could have a longer duration on ART. Compared to children who were diagnosed and initiated ART in the first year of life, children initiating ART between the first and the second years of life were 32% less likely to die (aHR 0.68 95% CI = 0.61–0.75), with lower hazard ratios in each older age group (Table 3). Compared to WHO clinical stage 1, children with WHO clinical stages 3 (aHR 10.6; 95% CI = 9.70–11.74) and 4 (aHR 16.49 95% CI = 14.56–18.67) had increased mortality (Table 3). The frailty coefficient was >0, showing that the survival rates differed by a factor of 2.18 across the health facilities.

**Table 3:** Multi-level Proportional hazard ratios for factors associated with mortality among CLHIV on ART in Zambia.

**Characteristic**	Unadjusted Hazard ratios (CI)^*^	Adjusted Hazard Ratios (CI)^**^	Adjusted Hazard ratios (Clustering at health facility level) (CI)^***^
**Sex**			
Female	1	1	1
Male	1.18 (1.11–1.27)	1.17 (1.09–1.25)	1.14 (1.06–1.23)
**Age when starting ART**			
Birth−1 year	1	1	1
1–2 years	0.74 (0.67–0.81)	0.74 (0.67–0.81)	0.68 (0.61–0.75)
3–5 years	0.31 (0.34–0.41)	0.31 (0.27–0.35)	0.35 (0.31–0.40)
6–9 years	0.29 (0.27–0.35)	0.29 (0.26- 0.33)	0.39 (0.34–0.45)
10–15 years	0.28 (0.26–0.33)	0.28 (0.25–0.32)	0.56 (0.48–0.64)
**WHO clinical staging**			
Stage 1	1	1	1
Stage 2	2.86 (2.52–3.24)	2.92 (2.58–3.31)	4.11 (3.60–4.68)
Stage 3	6.96 (6.38–7.59)	6.25 (5.72–6.82)	10.6 (9.70–11.74)
Stage 4	11.6 (10.4–13.0)	9.53 (8.48–10.72)	16.4 (14.56–18.67)
**Time from testing to ART initiation**			
Same day	1	1	1
One week	1.27 (1.10–1.46)	1.24 (1.08–1.43)	1.19 (1.01–1.39)
Up to 1 month	1.22 (1.12–1.34)	1.27 (1.16–1.39)	1.39 (1.24–1.55)
More than a month	0.77 (0.71–0.84)	0.89 (0.81–0.97)	1.18 (1.06–1.32)

* Tested using Cox proportional Hazards ratio.

** Hazards adjusted for age at ART start and sex.

*** Frailty model adjusting for clustering at facility level.

*The variability of survival between the health facilities 2.18 (95% CI 1.86–2.63)*.

Three separate models were used restricting the analysis to subsets of children with data for CD4 counts at initiation, WAZ and the first line drug regime used at ART initiation. All models were adjusted for sex, WHO clinical stage, and time on ART, and the estimates for these variables were similar to the full model although the significance of the effects was greatly reduced (Table 4). Higher mortality was observed in those who were severely malnourished (aHR 4.51, 95% CI 2.57–7.92) compared to those who were not severely malnourished. Higher values of frailty for the model including malnourishment indicates the additional clustering of malnourishment in health facilities, increasing the variability of the survival rates by 3.22 across the health facilities. In the same model, those who initiated ART with a CD4 count of <500 cells/μl (aHR 1.76, 95% CI 1.22–2.54) had higher mortality than those who initiated ART with a CD4 count >500 cells/μl (Table 4).

**Table 4:** Multi level adjusted and unadjusted hazards ratios for factors associated with mortality among CLHIV on ART in Zambia clustered at facility level.

**Characteristic**	**All children**	**Children with data on CD4 count at initiation**	**Children with data on WAZ at initiation**	**Children with data on first line drug regimen**
Total number analyzed	55,752	4,105	5,543	14,232
**Sex**				
Female	1	1	1	1
Male	1.14 (1.06–1.23)	1.27 (0.1.22–2.54)	1.12 (0.82–1.53)	1.10 (0.96–1.25)
**WHO clinical staging**				
Stage 1	1	1	1	1
Stage 2	4.18 (3.67–4.76)	3.11 (1.81–5.32)	5.88 (3.72–9.30)	7.02 (5.55–8.88)
Stage 3	11.7 (10.6–12.8)	8.12 (5.46–12.0)	10.6 (7.10–15.9)	29.2 (24.6–34.7)
Stage 4	18.7 (16.6–21.2)	13.3 (7.52–2.52)	11.5 (6.16–21.6)	61.9 (50.2–76.3)
**CD4 counts**				
>=350 (Normal)		1		
<350 (Low)		1.76 (1.22–2.54)		
**Weight for AGE—*****Z*****-score (WAZ)**				
Normal			1	
Moderate			3.22 (1.98–5.25)	
Severe			4.51 (2.57–7.92)	
**Drug regimen**				
NRTI based				1
EFV based				0.28 (0.22–0.35)
NVP based				1.17 (1.01–1.36)
**Assessment of clustering**				
Variability of survival between the facilities	2.18 (1.86–2.63)	2.18 (1.49–4.66)	3.10 (1.97–6.42)	1.77 (1.46–2.34)

*All models adjusted for sex and WHO clinical stage clustering at health facility level*.

### Estimation of Cumulative Hazards Curves of Death Among CLHIV on ART

**Figure 1:**
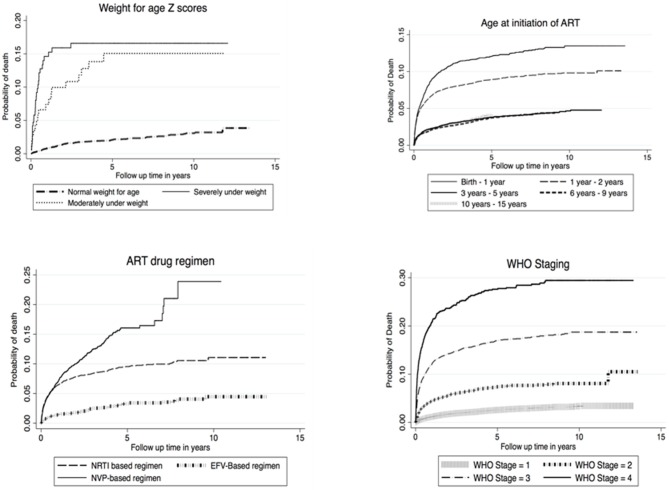
Nelson Aalen cumulative Hazard estimates.

Nelson Aalen cumulative Hazard estimates were analyzed for factors affecting survival in CLHIV on ART in Zambia and cumulative Hazard curves were derived. Factors analyzed included WHO staging, age at ART initiation, ART drug regimen and weight for age Z-scores. Overall cumulative hazards increased rapidly between the first and third year of follow up time across all factors that were estimated. The cumulative proportions of children who died on NVP based drug regimen was 15% higher than children who were on NRTI and EFV based regimen in the first 5 years of follow up time. Similar increases in mortality were also evident among children that initiated ART in WHO clinical stage 4, where steady increases of up to 30% were noticed for close to 10 years of follow up see Figure 1.

## Discussion

Our study outlines survival of children on ART over a 13 year period (2004–2017) in Zambia. Studies in the past showed that without ART, more than 50% of CLHIV would die before their second birthday (5). For all children, mortality rates at ART initiation were high but lower than those reported in CLHIV prior to ART availability. Overall the SmartCare database showed 3,265 children on ART died during the 13 years, which gave an overall estimate of 1.1 deaths per 100-person years.

Mortality rates in our study decreased with longer duration on ART. Mortality rates in children who had been on ART for 5 years or more were only slightly higher than those reported for HIV negative children in Zambia (23). The overall mortality rate of 1.1 per 100 person-years reported in this study changed with duration on ART. In the first year on ART, the mortality rate was 12 per 100 person-years, which reduced to 8 per 100 person-years between the first and second year, 1.6 per 100 between the second and fifth year, and 0.62 after the fifth year. Our mortality rates over the first 2 years on ART were higher than those reported in a 2 year follow up study in Addis Ababa, Ethiopia (1.2 per 100 person-years), a 1 year follow up study in Makurdi, Nigeria (3.0 per 100 person-years), and a 2 year follow up study in Eastern Ethiopia (2.1 per 100 years). However, our overall mortality rate was lower than those reported in a 7 year South African study (4.7 per 100 person-years) and an eight-year study in Jos, Nigeria (14, 16, 17). The variation in mortality rates could be due to larger sample size in our study compared to other studies used in the comparison. Another reason could be the year in which the study was conducted as WHO guidelines being used at that time could have affected time to ART initiation, drug regimen and in turn mortality (10, 14).

Children that initiated ART in Lusaka, Copperbelt, and Southern provinces as well as children that initiated ART in hospitals and health centers had higher rates of mortality in our study. Male children on ART in our study had increased risk of mortality in contrast to findings by Zanoni et al. (15), in a South African study where female child had increased chances of mortality compared to their male counterparts. Nonetheless, our findings are in line with findings by (16) where higher mortality rates was experienced among males (66.7%) than females (33.3%) as well as in a cohort of HIV uninfected children where male children had 2% higher mortality than female children sub Saharan Africa (24).

This study used a modified Cox regression to allow for differences in survival due to unobserved covariates at facility level. In these models, the estimated variance of health facility frailty terms was significantly >0, indicating large variability in the survival rates across facilities. The coefficient of variation for mortality across health facilities, showed mortality rates were more than twice as high (coefficient of variation = 2.18) in some facilities than the average. This difference could be due to varying resources in different facilities, training and availability of staff, and the quality of services. This high inequality needs to be addressed by the Ministry of Health to ensure equitable services to reduce HIV mortality for all Zambian CLHIV.

We also found that the risk of mortality was highest among infants and younger children initiated on ART, which is the period for highest risk of death for HIV negative children also. The risk of death reduced with increasing age. This could be due to non-adherence, sub optimal doses or exposure to perinatal antiretroviral therapy, which may lead to rapid disease progression and later death in infants and younger children (25). Another reason could also be that diagnosing HIV infection in infants is intricate and this can delay ART initiation in this age group (26). However, the proportion of children dying in this study did not account for over 50% of children analyzed in any age group showing improvements in survival compared to studies by Mulugeta et al. (5) and Tariro (6). Our study was in line with Studies in Asia and Africa that showed survival rates above 50% (84% to 97%) after 12 months of ART for children initiated on ART at a median age of 5–7 years, as well as another multi country study in Africa that also reported above 90% (93%) survival rates after 2 years of follow up on ART (18).

We found late initiation on to ART from time of HIV testing was predictive of reduced survival. Studies have shown that late presentation for early infant diagnosis (EID) services leads to poor prognosis (27, 28). This is also evidenced by findings of Bolton-Moore et al. (29) in earlier studies in Zambia. Our findings showed high mortality rates among children that initiated ART between 1 week and 1 month after HIV diagnosis. Current WHO guidelines call for same day ART initiation after HIV diagnosis which gives children the greatest benefit in survival (30). Our findings are in line with findings in a study done in South Africa that reported that 64% of deaths occurred within 3 months of ART initiation (15); a Nigerian study, where 81.3% and 84.4% of deaths occurred in the first 6 months and within 1 year, respectively (16) and an Ethiopian study that reported a high number of deaths within the first sixth months of starting ART (31, 32). This pattern of increased mortality could suggest that HIV-infected children presented with poor immunological or clinical states at ART initiation or that CLHIV require some time before the benefits of ART are fully realized, especially when ART is initiated early (13, 18, 33). However, our study showed reducing mortality with increase in time to ART initiation above 1 months. This is similar to findings in a 5-year cohort study in Thailand where children that initiated ART in infancy had higher risks of mortality compared to children that initiated ART at older ages (18). The cohorts of children initiating ART at older ages, probably reflects those who survived with HIV, and therefore reflect a “survivor effect” of the older children (18).

The SmartCare database showed 3,265 children on ART died over the 13 years, but some deaths could have been missed and not reported to the health facility staff. In the SmartCare data, CD4 counts, weight and ART regime were not routinely recorded for every child but may only be recorded when triggered by a clinical event. We did not attempt to impute these data but instead carried out subgroup analyses in those children where the data are recorded to assess whether risk factors differed in the subgroups. The measures of advanced HIV diseases, low CD4 count, severe acute malnutrition, and WHO clinical stages 3 and 4 were all associated with reduced survival and this is seen in our results (13, 15). These results are consistent with earlier an study in Zambia which showed that mortality was higher in children with lower WAZ scores (29).

This study was not without limitations. The main purpose of the SmartCare data is for clinical care, treatment and follow up of CLHIV in health facilities where staff are overburdened. In these busy health care facilities vital information such as laboratory results and weight were not collected at every visit, resulting in a large number of missing data, which meant these factors could not be included in our main analysis The results for these variables should be interpreted with caution, as they did not represent all children. However, the consistency of the demographic risk factors across the sub-group analysis is encouraging. The SmartCare database may have missed some deaths when they did not occur at the health facility, as these might not have been reported, or entered into the data base, which could underestimate the mortality in children on ART. Moreover, loss to follow up of children initiated on ART is common among children on ART in Zambia and has been documented in earlier studies (20, 34).

## Conclusions

Children with HIV in Zambia are surviving much longer than was predicted before ART was introduced 14 years ago. This key finding adds to the literature on analysis of survival in CLHIV in low income settings like Zambia. However, survival was dependent on the age at which ART was initiated, the time on ART, type of health care facility used at ART initiation and the province where services were accessed. This highlights the need to increase investments in early infant diagnosis (EID) and make services more equitable across Zambia to ensure timely HIV testing and ART initiation for CLHIV.

## Data Availability Statement

The Zambian Ministry of Health has the sole authority over the dataset that was used in this study, thus, it cannot be shared online. Any further information or interest to use the data should be addressed to the Zambian Ministry of Health (www.moh.gov.zm).

## Author Contributions

TM, JT, and CM participated in the conception of the study, co-ordination, acquisition of data and drafted the manuscript. TM and JT carried out the statistical analysis. CM, PM, JT, and TM reviewed all the content related to interpretation of the findings and participated in the critical review and editing of the manuscript drafts for scientific merit and depth. All authors read and approved the final manuscript.

### Conflict of Interest

The authors declare that the research was conducted in the absence of any commercial or financial relationships that could be construed as a potential conflict of interest. The handling editor declared a shared affiliation, though no other collaboration, with several of the authors JT and PM within the last 2 years, at time of review.

## References

[1] UNAIDS FACT SHEET – WORLD AIDS DAY 2019 GLOBAL HIV STATISTICS. 2019, UNAIDS (2019).

[2] UNICEF Children, HIV and AIDS: The World Today and in 2030 - UNICEF DATA. (2018). Available online at: https://data.unicef.org/resources/children-hiv-and-aids-2030/ (accessed March 19, 2020).

[3] UNAIDS Country Factsheet, Zambia 2018. (2018). Available online at: https://www.unaids.org/en/regionscountries/countries/zambia (accessed March 19, 2020).

[4] Avert HIV and AIDS in Zambia AVERT. (2017). Available Online at https://www.avert.org/professionals/hiv-around-world/sub-saharan-africa/zambia (accessed March 19, 2020).

[5] Mulugeta A Henok A Tewelde T Dube L Determinants of survival among HIV positive research article open access children on antiretroviral therapy in public hospitals, addis ababa, ethiopia. Qual Prim Care. (2017) 25:235–41. 10.1186/s12889-019-6482-1

[6] Tariro MA Clinical Outcomes in Children and Adolescents with Chronic HIV Infection in Zimbabwe: Preliminary Report of the PAP1 Study. Results. USAID (2014). Available online at: https://aidsfree.usaid.gov/sites/default/files/aidstar-one_clinical_outcomes_children_adolescents_hiv_zimbabwe.pdf (accessed March 19, 2020).

[7] Kabue MM Buck WC Wanless SR Cox CM McCollum ED Caviness AC. Mortality and clinical outcomes in HIV-infected children on antiretroviral therapy in Malawi, Lesotho, and Swaziland. Pediatrics. (2012) 130:e591–9. 10.1542/peds.2011-1187 22891234 PMC3962849

[8] Ebissa G Deyessa N Biadgilign S. Predictors of early mortality in a cohort of hiv-infected children receiving high active antiretroviral treatment in public hospitals in ethiopia. AIDS Care. (2015) 27:723–30. 10.1080/09540121.2014.997180 25599414

[9] Davies MA Gibb D Turkova A. Survival of HIV-1 vertically infected children. Curr Opin HIV AIDS. (2016) 11:455–64. 10.1097/COH.0000000000000303 27716730 PMC5384722

[10] Davies MA Phiri S Wood R Wellington M Cox V Bolton-Moore C. Temporal trends in the characteristics of children at antiretroviral therapy initiation in Southern Africa: the IeDEA-SA collaboration. PLoS ONE. (2013) 8:e81037. 10.1371/journal.pone.0081037 24363808 PMC3867284

[11] Violari A Cotton MF Gibb DM Babiker AG Steyn J Madhi SA. Early antiretroviral therapy and mortality among HIV-infected infants. N England J Med. (2008) 359:2233–44. 10.1056/NEJMoa0800971 19020325 PMC2950021

[12] Luzuriaga K Mcmanus M Mofenson L Britto P Graham B Sullivan JL. A trial of three antiretroviral regimens in HIV-1–infected children. New England J. Med. (2004) 350:2471–80. 10.1056/NEJMoa032706 15190139

[13] Edessa D Asefa F Sheikahmed J Early mortality among HIV-positive children initiated anti-retroviral therapy in eastern ethiopia: a retrospective cohort study. Sci Technol Arts Res J. (2016) 4:157 10.4314/star.v4i2.19

[14] Biru M Hallström I Lundqvist P Jerene D. Rates and predictors of attrition among children on antiretroviral therapy in ethiopia: a prospective cohort study. PLoS ONE. (2018) 13:e0189777. 10.1371/journal.pone.0189777 29408897 PMC5800538

[15] Zanoni BC Phungula T Zanoni HM France H Feeney ME. Risk factors associated with increased mortality among HIV infected children initiating antiretroviral therapy (ART) in South Africa. PLoS ONE. (2011) 6:e22706. 10.1371/journal.pone.0022706 21829487 PMC3146475

[16] Ebonyi AO Oguche S Meloni ST Sagay SA Kyriacou DN Achenbach CJ. Predictors of mortality in a clinic cohort of HIV-1 infected children initiated on antiretroviral therapy in Jos, Nigeria. J AIDS Clin Res. (2014) 5:403. 30416842 10.4172/2155-6113.1000403 PMC6223308

[17] Anigilaje EA Aderibigbe SA. Mortality in a Cohort of HIV-infected children: a 12-month outcome of antiretroviral therapy in makurdi, Nigeria. Adv Med. (2018) 2018:6409134. 10.1155/2018/6409134 30018988 PMC6029505

[18] Collins IJ Jourdain G Hansudewechakul R Kanjanavanit S Hongsiriwon S Ngampiyasakul C. Long-term survival of HIV-infected children receiving antiretroviral therapy in Thailand: a 5-year observational cohort study. Clin Infect Dis. (2010) 51:1449–57. 10.1086/657401 21054181 PMC3106246

[19] MOH E-Health Strategy 2013-2016. Lusaka: MOH (2013).

[20] Sutcliffe CG Bolton-Moore C van Dijk JH Cotham M Tambatamba B Moss WJ. Secular trends in pediatric antiretroviral treatment programs in rural and urban Zambia: a retrospective cohort study. BMC Pediatrics. (2013) 10:54. 10.1186/1471-2431-10-54 20673355 PMC2919522

[21] MOH Zambia Cosolidated Guidelines for Treatment and Prevention of HIV Infection. Ministry of Health (MOH). Lusaka: Department of Clinical Care and Department of Public Health (2017).

[22] WHO WHO Growth Standards: Length / Height for Age, Weight for Age, Weight for Length, Weight for Height and Body Mass Index for Age: Methods and Development. WHO (2006).

[23] C S. O., MoH, and ICF Zambia Demographic and Health Survey 2018: Key Indicators. Lusaka: Central Statistical Office (2018). Available online at: https://www.zamstats.gov.zm/phocadownload/Demography/ZDHS%20Key%20Indicator%20Report%202018.pdf (accessed March 19, 2020).

[24] Sawyer CC. Child mortality estimation:estimating sex differences in childhood mortality since1970. PLoS ONE. (2012) 9:e1001287. 10.1371/journal.pmed,.1001287 22952433 PMC3429399

[25] Kabue MM Kekitiinwa A Maganda A Risser JM Chan W Kline MW. Growth in HIV-infected children receiving antiretroviral therapy at a pediatric infectious diseases clinic in Uganda. AIDS Patient Care STDs. (2008) 22:245–51. 10.1089/apc.2007.0049 18298315

[26] Kankasa C Carter RJ Briggs N Bulterys M Chama E Cooper ER Routine offering of HIV testing to hospitalized pediatric patients at university teaching hospital, Lusaka, Zambia: acceptability and feasibility. J Acquir Immune Defic Syndr. (2009) 25:202–8. 10.1097/QAI.0b013e31819c173f PMC5117627 19504732

[27] Koye DN Ayele TA Zeleke BM. Predictors of mortality among children on antiretroviral therapy at a referral hospital, Northwest Ethiopia: a retrospective follow up study. BMC Pediatrics. (2012) 12:161. 10.1186/1471-2431-12-161 23043325 PMC3478986

[28] Sidamo NB Hebo SH Original article survival time and its predictors among hiv-infected children after antiretroviral therapy in public health facilities of arba minch town, gamo gofa zone, southern ethiopia. Ethiopian J Health Dev. (2018) 32:88–96. 10.4172/2090-7214.1000267

[29] Bolton-Moore C Mubiana-Mbewe M Cantrell RA Chintu N Stringer EM Sinkala M. Clinical outcomes and CD4 cell response in children receiving antiretroviral therapy at primary health care facilities in zambia. JAMA. (2007) 298:1888–99. 10.1001/jama.298.16.1888 17954540

[30] WHO (2017). Guidelines for Managing Advanced HIV Disease and Rapid Initiation of Antiretroviral Therapy. Geneva: World Healh Organisation Available online at: http://apps.who.int/iris/bitstream/handle/10665/255884/9789241550062-eng.pdf?sequence=1 (accessed March 19, 2020) 29341560

[31] Kedir AA Desta A Fesseha G Factors affecting survival of HIV positive children taking antiretroviral therapy at adama referral hospital and medical college, ethiopia. J AIDS Clin Res. (2014) 5:1–6. 10.4172/2155-6113.1000289

[32] Taye B Shiferaw S Enquselassie F. The impact of malnutrition in survival of HIV infected children after initiation of antiretroviral treatment (ART). Ethiop Med J. (2010) 48:1–10. 20607992

[33] Bong CN Yu JK Chiang HC Huang WL Hsieh TC Schouten EJ Risk factors for early mortality in children on adult fixed-dose combination antiretroviral treatment in a central hospital in Malawi. AIDS. (2007) 20:1805–10. 10.1097/QAD.0b013e3282c3a9e4 17690580

[34] Singh J Filteau S Todd J Gumede-Moyo S. Progress in the performance of HIV early infant diagnosis services in zambia using routinely collected data from 2006 to 2016. BMC Public Health. (2018) 18:1297. 10.1186/s12889-018-6222-y 30477465 PMC6258281

